# Quantitative evaluation of the medical resource allocation policy in China: a PMC-index model approach

**DOI:** 10.1186/s12962-026-00788-z

**Published:** 2026-06-25

**Authors:** Wanwen Jia, Yitian Shao

**Affiliations:** 1 The New Type Key Think Tank of Zhejiang Province “China Research Institute of Regulation and Public Policy”, Zhejiang University of Finance and Economics, No. 18 Xueyuan Street, Hangzhou, Zhejiang 310018 China; 2 China Institute of Regulation Research, Zhejiang University of Finance and Economics, No. 18 Xueyuan Street, Hangzhou, Zhejiang 310018 China; 3 Zhejiang Province Key Think Tank: Institute of Ecological Civilization, Zhejiang A&F University, No. 666 Wusu Street, Hangzhou, Zhejiang 311300 China

**Keywords:** Medical resource allocation policy, PMC-index model, Policy evaluation, Text-mining

## Abstract

**Background:**

A comprehensive policy framework constitutes a crucial institutional foundation for the sustainable development of healthcare systems and quantitative evaluation of public policies offers a scientific basis for policy refinement and optimization. Amid ongoing healthcare reform and profound shifts in the structure of demand for medical resources, systematically assessing the textual quality of national-level medical resource allocation policies, together with examining of their structural characteristics and evolutionary trajectories, carries considerable practical significance.

**Methods:**

Through a systematic screening process, 16 national-level policy documents on medical resource allocation were identified. Text-mining techniques were applied to extract high-frequency keywords and build a keyword co-occurrence network, after which the PMC-index model was then used to quantitatively assess the overall quality of these policies. Heterogeneity analyses were then conducted across policy quality grades and time periods to reveal structural differences and evolutionary patterns.

**Results:**

The average PMC-index across the 16 policies was 6.67, suggesting that China’s medical resource allocation policies are generally of relatively high quality. However, the analysis revealed several structural weaknesses, including limited predictive and forward-looking content, insufficient medium- to long-term planning, and an under-supply of higher-level policy instruments. Temporal analysis showed that the PMC-index increased from the Exploration Stage to the Deepening Stage before declining modestly in the Focus Stage.

**Conclusions:**

China’s medical resource allocation policies are of relatively high overall quality, yet further improvements remain necessary. Future policy efforts should focus on three priorities: optimizing the hierarchical structure of the policy system, strengthening policy predictability and medium- to long-term planning orientation, and refining policy content and implementation mechanisms. Together, these efforts would enhance the systematization, stability, and governance effectiveness of the policy framework.

**Supplementary information:**

The online version contains supplementary material available at https://doi.org/10.1186/s12962-026-00788-z.

## Introduction

The Chinese government has consistently placed the healthcare sector at the strategic core of national development. After decades of reform and institutional restructuring, the overall scale of medical resources in China has expanded steadily, while healthcare service delivery capacity has improved markedly. As a foundational element for safeguarding public health and advancing high-quality development in the health sector, the quantity, structural composition, and spatial distribution of medical resources are critical determinants of both resource utilization efficiency and population health outcomes.

However, existing evidence suggests that China’s healthcare system continues to experience structural mismatches between medical resource allocation and the actual healthcare needs of patients [1–3]. In 2022, for example, the distribution of health technicians across primary, secondary, and tertiary hospitals stood at 7.17%, 35.75%, and 57.08%, respectively. This pattern reveals a pronounced concentration of medical human resources in higher-level institutions, which is not well aligned with the demand for services related to common and frequent diseases, many of which can be more appropriately managed at the primary care level [4]. In addition, prior studies report that approximately 64.8% of outpatients with chronic diseases and 61.6% of inpatients at large public hospitals could in principle be referred to secondary hospitals or primary healthcare institutions [5]. At the same time, substantial regional disparities persist in medical resource distribution, underscoring the long-standing challenge of geographic imbalance [6, 7].

To address these challenges, the Chinese government has introduced a series of policy measures aimed at optimizing the allocation and improving the utilization efficiency of medical resource. Since the launch of a new round of healthcare system reforms in 2009, a wide range of policy documents have been issued, covering the regulation, structural optimization, and spatial redistribution of medical resources. These include overarching reform guidelines, the 13th and 14th Five-Year Health Development Plans, and targeted initiatives such as the development of medical consortia, county-level healthcare alliances, and compact urban healthcare groups. Together, these policies have gradually established a comprehensive policy framework addressing both the supply and demand sides of medical resource allocation.

At the macro-policy level, both the 2024 Central Economic Work Conference and the 2025 Government Work Report identified the enhancement of healthcare service capacity as a central priority, with initiatives such as the “Strengthening Primary Healthcare Infrastructure Initiative” and “Promoting the Downward Allocation of Medical Resources.” This emphasis reflects the state’s sustained commitment to reallocating medical resources toward lower-level institutions and strengthening grassroots healthcare capacity, while highlighting the urgency and strategic importance of optimizing medical resource allocation. A systematic review and quantitative assessment of national-level medical resource allocation policies thus carry substantial theoretical and practical value, contributing to a deeper understanding of the underlying policy logic, the identification of existing gaps, and the enhancement of policy effectiveness.

Against this backdrop, this study takes national-level policy documents directly related to medical resource allocation as its primary subjects of analysis. It seeks to address the following core research questions: What is the overall quality of China’s national-level medical resource allocation policies? What structural characteristics and potential weaknesses do these policies exhibit in terms of policy nature, policy instruments, and policy content? Do the policies display significant structural heterogeneity, and what evolutionary patterns emerge over time?

This study makes three main contributions to the existing literature. First, in scope, it offers a systematic quantitative evaluation of national-level medical resource allocation policies in China, thereby broadening healthcare policy research into a relatively underexplored domain. Second, methodologically, this study goes beyond a routine application of the PMC-index model to a new set of policy documents. Rather, it integrates text mining into the identification and refinement of evaluation dimensions, constructing a more policy-sensitive analytical framework for indicator design and quantitative evaluation. Third, in analytical and practical value, this study extends policy evaluation from static assessment to a dynamic and heterogeneous perspective. By examining policy differences across quality grades and temporal stages, it uncovers the structural characteristics and evolutionary trajectory of China’s medical resource allocation policies, offering a more comprehensive basis for policy optimization and for improving medical resource allocation efficiency.

The remainder of the study is organized as follows. Section “Literature review” reviews the relevant literature and presents the conceptual framework of the study. Section “Methods” describes the data and methodology. Section “Results” presents the results of the quantitative policy evaluation, with an in-depth analysis of policy heterogeneity and temporal evolution. Section “Conclusions and policy implications” summarizes the main findings and proposes policy recommendations.

## Literature review

### Research on the evaluation of medical resource allocation policies

Existing studies have systematically examined medical resource allocation, focusing primarily on equity, allocation structure, and efficiency. One strand of literature has employed quantitative indicators such as the Gini coefficient and the Theil index to assess disparities in medical resource distribution and the level of equity. For instance, Chai et al. (2024) examined the equity of China’s medical human resources from both demographic and geographic perspectives, finding that although overall equity has improved, pronounced regional imbalances persist [8]. Zhou (2025) focused on public health resources and analyzed their equity and efficiency [9]. Another strand has turned its analytical focus toward the efficiency of medical resource allocation [10] or evaluated the effects of relevant policies on allocation efficiency [11, 12]. Taken together, the existing literature generally agrees that China’s medical resource allocation efficiency remains suboptimal with considerable room for improvement [13, 14], while inequities in medical service utilization continue to exist [15].

This studies primarily focus on outcome-oriented measures, offering in-depth characterizations of the current status and effectiveness of medical resource allocation. Such analyses provide important empirical evidence for identifying structural problems within the medical resource allocation system. However, systematic quantitative research on the design quality, internal structural consistency, and policy-instrument configurations of medical resource allocation policies based on policy texts remains relatively limited. In particular, little attention has been paid to the internal rationality of national-level medical resource allocation policies or to how their design quality can be systematically assessed.

Only a few exploratory studies have turned their analytical focus to policy texts. For instance, Cui et al. (2022) conducted a quantitative analysis of medical resource allocation policy texts related on medical consortia from the perspectives of policy keywords and policy instruments, identifying significant disparities in the use of different types of policy instruments, particularly the relative underuse of demand-driven and stock-adjustment instruments [16]. Although these studies have begun to reveal the design characteristics of medical resource allocation policies, considerable scope remains for further research, particularly in broadening evaluation dimensions and conducting systematic dynamic comparisons across policies and over time.

### Quantitative evaluation of healthcare policy texts

In recent years, the quantitative evaluation of policy texts has emerged as an important analytical approach for examining the progress of healthcare system reform, accompanied by the rapid development of related methodologies and applied research. Researchers typically combine qualitative and quantitative methods to systematically review and compare healthcare policies. Existing studies have examined a wide range of policy areas, including hierarchical medical system policies [17, 18], the inclusion of assisted reproductive technologies in medical insurance [19], commercial health insurance policies [20], internet-based medical service policies [21], digital health policies [22], and public hospital reform policies [23, 24]. Together, this body of research offers valuable methodological insights for the quantitative analysis of healthcare policy texts.

Building on this foundation, a growing body of research has applied the PMC-index (Policy Modeling Consistency Index) to the quantitative evaluation of medical policies. The PMC-index model constructs a multidimensional indicator system to comprehensively score and visually present policy texts across multiple dimensions, offering several advantages: flexible dimension configuration, transparent weighting procedures, and intuitive presentation of evaluation results. Originally proposed by Estrada to assess policy modeling consistency, the model has since been widely applied in policy evaluation research [25, 26]. Within the medical policy domain, existing studies have employed the PMC-index model to evaluate various policy areas. For example, Yang et al. (2022) conducted a quantitative assessment of health promotion policies in Sichuan Province [27], while Yang et al. (2022) examined traditional Chinese medicine development policies through a PMC-index analysis [28]. Fu et al. (2025) conducted a quantitative PMC-index evaluation of China’s hierarchical medical system policy and further integrated a Logit model to explore the relationship between policy formulation and implementation [17].

Some scholars have integrated text-mining techniques with the PMC-index model to strengthen the empirical basis for indicator construction, primarily through methods such as keyword extraction and topic identification. Zhai et al. (2022) combined text mining with the PMC-index model to analyze the strengths and weaknesses of telemedicine policies and to identify optimization pathways [29]. Zhang and Xiang (2023) focused on healthcare security fund regulation policies, constructing a PMC-index model for quantitative evaluation based on policy content mining [30]. Duan et al. (2024) systematically examined the internal consistency and evolutionary characteristics of China’s digital healthcare policies from a developmental perspective [31]. In addition, Guo et al. (2024) and Gong et al. (2025) applied comparable analytical frameworks to the evaluation of medicine innovation policies and two-way referral policies, respectively [32], [33]. Chen et al. (2025) and Li et al. (2025) further extended this integrated methodology to smart elderly care services and integrated elderly care and medical services, respectively [34, 35].

In summary, although existing studies provide valuable references for the quantitative evaluation of medical resource policy texts, considerable scope for further development remains. First, in research subjects, prior studies have predominantly focused on specific policy domains such as healthcare security fund regulation, digital healthcare, and hierarchical medical system policy; systematic quantitative evaluations focusing on the quality of national-level medical resource allocation policy texts remain relatively limited. Second, methodologically, although an increasing number of studies have attempted to integrate text-mining techniques with the PMC-index model, some evaluations still rely heavily on empirically constructed indicator systems without sufficiently engaging with the substantive content of policy texts. Third, in research content, existing applications of the PMC-index model remain largely static, with relatively limited systematic comparative research examining structural heterogeneity across different policies or their evolutionary patterns over time.

**Fig. 1:**
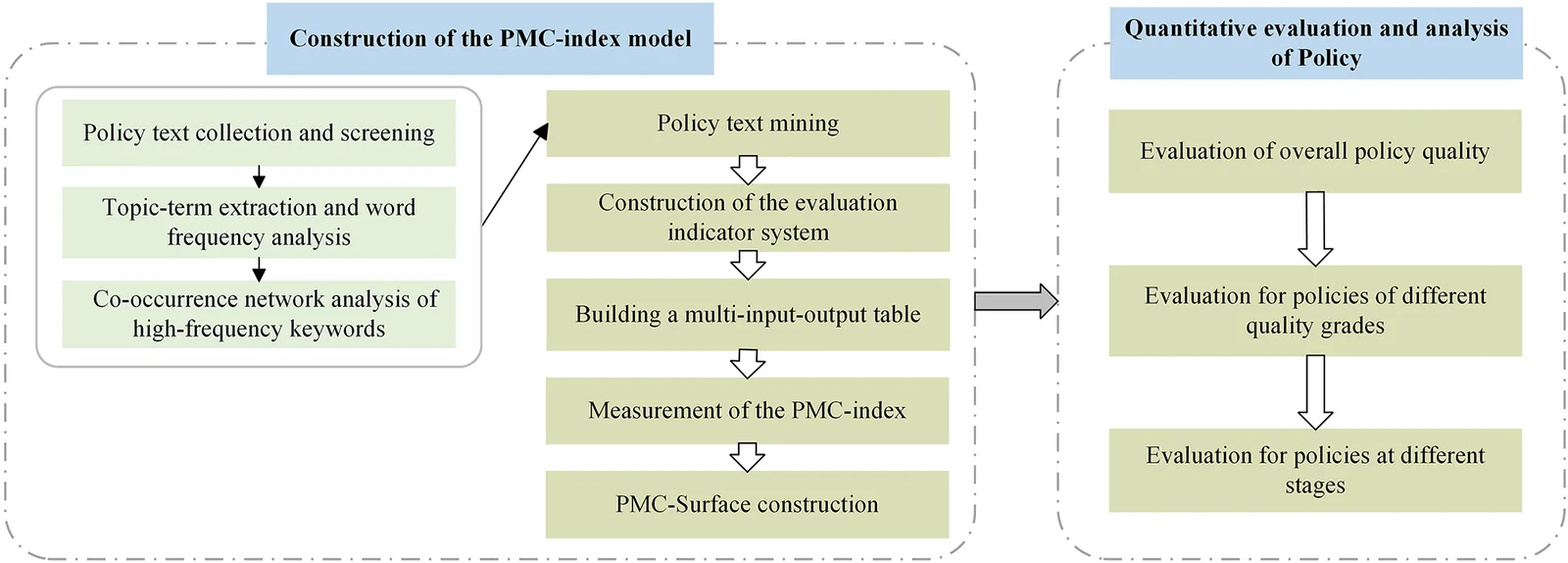
Research framework

To address these gaps, this study takes national-level medical resource allocation policy texts as its primary research objects. By integrating policy text-mining techniques with the PMC-index model, it constructs an evaluation indicator system consisting of nine primary variables and 41 secondary variables and applies it to a quantitative assessment of 16 representative policies. The study not only evaluates the overall quality of medical resource allocation policies but also conducts heterogeneity analyses along two dimensions, namely policy quality grades and time periods, to identify structural differences and evolutionary characteristics across policies. These findings provide empirical evidence for improving China’s medical resource allocation policy framework. The research framework is illustrated in Fig. 1.

## Methods

### Data sources and sample selection

This study examines national-level policies concerning medical resource allocation. Since a new round of healthcare system reforms was launched in 2009, the study period spans 2009 to 2025. Policy documents were collected and processed as follows.

First, this study conducted a comprehensive keyword search using terms such as “medical resources,” “medical resource allocation,” “healthcare resources,” “medical and health resources,” “healthcare resource allocation,” “downward allocation of medical resources,” and “high-quality medical resources.” Policy documents were retrieved from the Peking University Legal Database, the official Chinese Government website, the State Council policy document repository, and the National Health Commission website. The retrieved documents were then screened based on their relevance to the research topic and the standardization of policy text formats. Only formal policy documents, including outlines, notices, and opinions, that explicitly addressed medical resource allocation were retained, yielding in an initial sample of 36 policy texts.

Second, the initially identified documents were further screened to improve comparability and representativeness. Documents were excluded if they (1) were only marginally related to medical resource allocation, meaning that medical resource allocation was not a principal policy objective, was mentioned only briefly or incidentally, and did not constitute a sufficiently substantive and self-contained textual basis for PMC-index coding; (2) belonged to non-substantive or non-standardized policy types and therefore lacked a sufficiently complete and stable textual basis for PMC-index coding; or (3) substantially overlapped with policies already included in the sample. Through this process, 20 documents were excluded, and 16 policies were ultimately retained as the final research sample (see Table 1). To enhance transparency and reproducibility, the excluded policy documents and the specific reasons for their exclusion are provided in the Supplementary Material. For lengthy policy documents covering broad topics, only the sections directly related to medical resource allocation were extracted for text analysis, minimizing potential interference from irrelevant content in the quantitative evaluation (e.g., P7).

**Table 1:** Policy texts on medical resource allocation

Code	Policy Title	Issuing Agency	Date Issued
P1	Opinions on Deepening the Reform of the Medical and Healthcare System	Central Committee of the Communist Party of China; the State Council	2009.03.17
P2	Notice on Issuing the Plan and Implementation Scheme for Deepening the Reform of the Medical and Healthcare System during the 12th Five-Year Plan Period	the State Council	2012.03.14
P3	Notice on Issuing the 12th Five-Year Plan for the Development of the Health Sector	the State Council	2012.10.08
P4	Notice on Issuing the National Plan for the Development of the Healthcare Service System (2015–2020)	General Office of the State Council	2015.03.16
P5	Implementation Opinions on Advancing Comprehensive Reform of County-Level Public Hospitals Nationwide	General Office of the State Council	2015.04.23
P6	Guiding Opinions on Promoting the Development of the Hierarchical Medical System	General Office of the State Council	2015.09.08
P7	Outline of the “Healthy China 2030” Plan	Central Committee of the Communist Party of China, the State Council	2016.10.25
P8	Notice on Issuing the 13th Five-Year Plan for Health and Wellness	the State Council	2016.12.27
P9	Notice on Issuing the 13th Five-Year Plan for Deepening the Reform of the Medical and Healthcare System	the State Council	2016.12.27
P10	Guiding Opinions on Promoting the Establishment and Development of Medical Consortia	General Office of the State Council	2017.04.23
P11	Notice on Issuing the Key Tasks for Deepening the Reform of the Medical and Healthcare System in 2019	General Office of the State Council	2019.05.23
P12	Notice on Issuing the Key Tasks for Deepening the Reform of the Medical and Healthcare System in 2021	General Office of the State Council	2021.05.24
P13	Notice on Issuing the Key Tasks for Deepening the Reform of the Medical and Healthcare System in 2022	General Office of the State Council	2022.05.04
P14	Opinions on Further Improving the Medical and Healthcare Service System	General Office of the Central Committee of the Communist Party of China; General Office of the State Council	2023.03.23
P15	Notice on Further Improving Mechanisms to Promote the Downward Allocation of Urban Medical Resources to County Hospitals and Primary-Level Institutions	National Health Commission; National Administration of Traditional Chinese Medicine; National Disease Control and Prevention Administration	2024.04.28
P16	Approval of the Implementation Plan for the Initiative to Strengthen Primary-Level Healthcare Capacity	the State Council	2025.09.07

### Construction of the PMC-index model

#### Variable identification through policy text mining

This section applies the text-mining software ROST CM 6 (ROST Content Mining System) to conduct a content analysis of the policy texts, identifying core themes and high-frequency keywords that serve as the foundation for defining the primary and secondary variables.

The policy texts were organized and imported into the ROST CM 6 database. Using its word segmentation and frequency analysis functions, core concepts and high-frequency keywords related to medical resource allocation were extracted from the policy samples. To enhance the validity of the results, terms lacking substantive policy meaning were excluded, including generalized verbs such as “play a role,” “encourage,” and “develop,” as well as high-frequency function words such as “aspect,” “continuously,” and “main.” Given synonymous expressions and lexical variations across the policy texts, semantically similar terms were consolidated. For example, “medical and health resources” and “health resources” were standardized “medical resources,” while “yiliao lianheti” (medical consortium) and its abbreviation “yilian-ti” were merged into “yilian-ti (medical consortium).” The final word frequency statistics for the medical resource allocation policy texts are presented in Table 2, listing the top 60 most frequent terms.

**Table 2:** Term frequency statistics

Topic Term	Frequency	Topic Term	Frequency	Topic Term	Frequency
Service	687	Primary-level	151	Medical resource	98
Health	630	Resource	144	Township health center	93
Medical	567	The public	144	Residents	92
Healthcare	561	Diagnosis and treatment	132	Performance	89
Hospitals	440	Patient	129	Chronic disease	89
Mechanism	394	Price	125	Rural area	88
Reform	368	Regions	124	Reform of the medical and healthcare system	87
System	303	Overall planning	120	Basic healthcare insurance	87
Public hospital	269	Pilot	120	Healthcare institution	86
Policy	246	Assessment	118	At all levels	82
Safeguard	238	Training	116	Hierarchical medical	80
Technology	198	Supervision	115	Referral	80
Primary healthcare institution	190	Insurance	110	Investment	80
Government	187	Medical consortium	109	Urban and rural	79
Healthcare insurance	185	Division of responsibilities	107	Medical and health	76
Regulation	185	Physician	106	Optimization	72
Public health	183	Innovation	106	Medicine and pharmaceutical	72
Medical service	180	Urban	106	Support	71
Doctor	168	County-level public hospital	104	Healthcare reform	70
Quality	155	Demand	100	Layout	68

As shown in Table 2, China’s current medical resource allocation policies concentrate on two main directions. First, the frequent occurrence of keywords such as “primary healthcare institution,” “primary level,” “county-level public hospital,” and “township health center” suggests that ongoing reforms prioritize strengthening the service capacity of primary healthcare institutions. Second, the clustered appearance of keywords such as “medical consortium,” “division of responsibilities,” “hierarchical medical,” “referral,” and “support” indicates that the primary implementation pathway is to optimize medical resource allocation and strengthen collaboration among medical institutions at all levels through mechanisms such as medical consortia and grassroots support, thereby advancing the implementation of the hierarchical medical system.

**Fig. 2:**
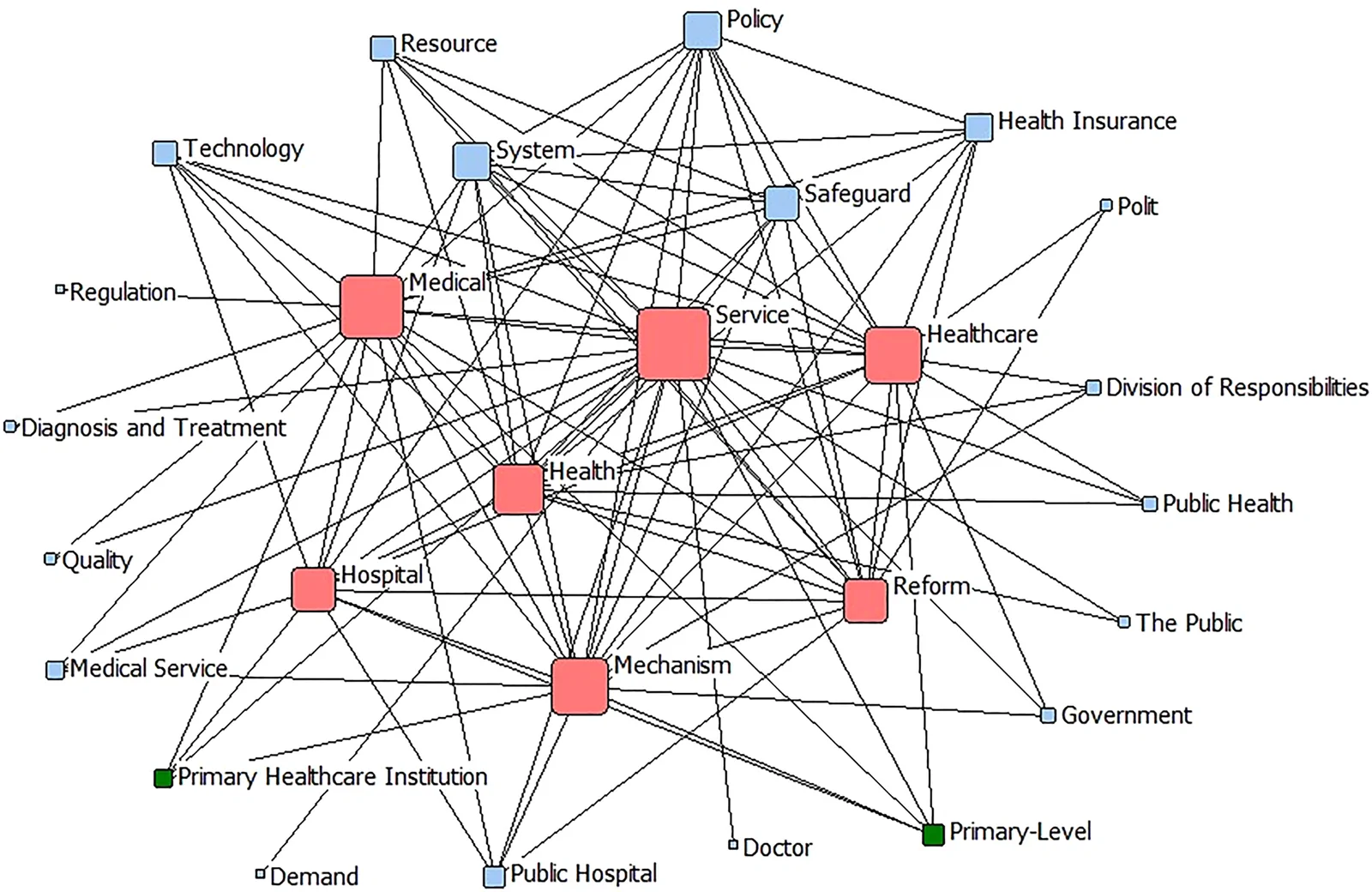
Co-occurrence network of high-frequency keywords

This study further employs social network analysis to construct a co-occurrence network of high-frequency policy terms, illustrating the relationships among policy themes. Using ROST CM 6 and NetDraw, the 16 policy texts were systematically analyzed, and a social network diagram of policy keywords was generated based on degree centrality (Fig. 2), visualizing the focal points of policy content. Degree centrality reflects the importance of policy themes: keywords with greater significance and stronger cohesion within the network appear as larger nodes. As shown in Fig. 2, the core keywords in medical resource allocation policies include “service,” “medical,” “healthcare,” “health,” “mechanism,” and “reform.” In addition, keywords such as “primary healthcare institution” and “primary level” occupy prominent positions, further confirming that policy development emphasizes the strengthening of primary healthcare, which is consistent with the word frequency statistics in Table 2.

#### Evaluation indicator system construction and multi-input-output table

A scientifically constructed evaluation indicator system is essential for conducting quantitative policy evaluation using the PMC-index model. In this study, the indicator system was developed by integrating the general logic of the PMC-index framework with the specific characteristics of medical resource allocation policies. Specifically, the framework design was informed by the policy text-mining results, particularly the high-frequency keywords and keyword co-occurrence patterns extracted from the policy texts. The construction also drew on the PMC-index framework proposed by Estrada (2011) [26] and prior studies on healthcare policy text evaluation [17, 29–31]. Building on these foundations, this study established a PMC-index evaluation indicator system for medical resource allocation policies, comprising nine primary variables and 41 secondary variables, as shown in Table 3.

**Table 3:** Evaluation indicator system of the PMC-index model

Primary Variable	Secondary Variable	Evaluation Criteria	Scoring Rule	References
X1 Policy Nature	X1:1 Prediction	Whether the policy reflects the prediction	N: [0, 1]	[29, 30]; policy text mining results
	X1:2 Supervision	Whether the policy reflects the supervision	N: [0, 1]	
	X1:3 Suggestion	Whether the policy reflects the suggestion	N: [0, 1]	
	X1:4 Guidance	Whether the policy reflects the guidance	N: [0, 1]	
	X1:5 Description	Whether the policy reflects the description	N: [0, 1]	
	X1:6 Support	Whether the policy reflects the support	N: [0, 1]	
X2 Policy Content	X2:1 Optimisation of medical resource allocation	Whether the policy addresses the optimization, downward allocation, or sharing of medical resources	N: [0, 1]	policy text mining results
	X2:2 Primary care service capacity	Whether the policy aims to improve the service capacity or quality of primary healthcare institutions	N: [0, 1]	
	X2:3 Division of labour and collaboration among medical institutions	Whether the policy strengthens mechanisms for division of labour and collaboration across healthcare institutions at all levels	N: [0, 1]	
	X2:4 Construction of hierarchical medical system	Whether the policy involves systems for differentiating acute and chronic conditions or standardizing treatment-seeking behavior	N: [0, 1]	
	X2:5 Management of public hospital expansion	Whether the policy addresses the control of excessive expansion of large public hospitals	N: [0, 1]	
	X2:6 Differentiated healthcare insurance payment mechanism	Whether the policy establishes differentiated insurance payment mechanisms across different levels of medical institutions	N: [0, 1]	
X3 Policy Objective	X3:1 Promoting healthcare equity	Whether the policy emphasizes the accessibility of healthcare in rural areas, primary-level regions, or for vulnerable groups	N: [0, 1]	policy text mining results
	X3:2 Improving efficiency of healthcare services	Whether the policy proposes to enhance the efficiency of healthcare resource utilization or service delivery	N: [0, 1]	
	X3:3 Advancing healthcare system reform	Whether the policy aims to introduce new mechanisms, models, or optimize existing systems (e.g., medical consortia)	N: [0, 1]	
	X3:4 Enhancing population health	Whether the policy explicitly aims to improve population health or promote health equity	N: [0, 1]	
X4 Policy Target	X4:1 Health administrative departments	Whether the policy involves health administrative departments	N: [0, 1]	[31]; policy text mining results
	X4:2 Hospitals	Whether the policy involves hospitals	N: [0, 1]	
	X4:3 Patients	Whether the policy involves patients (e.g., guidance on medical treatment-seeking behavior)	N: [0, 1]	
	X4:4 Medical staff	Whether the policy involves medical staff	N: [0, 1]	
X5 Policy Instruments	X5:1 Supply-side	Whether the supply-side policy instruments are applied in the policy	N: [0, 1]	[32, 36]
	X5:2 Demand-side	Whether the demand-side policy instruments are applied in the policy	N: [0, 1]	
	X5:3 Environmental-side	Whether the environmental-side policy instruments are applied in the policy	N: [0, 1]	
X6 Policy Timeliness	X6:1 Long term	Whether the policy content involves more than 5 years	N: [0, 1]	[30–32]
	X6:2 Medium term	Whether the policy content involves medium term (3–5 years)	N: [0, 1]	
	X6:3 Short term	Whether the policy content involves (1–3 years)	N: [0, 1]	
	X6:4 Within 1 year	Whether the policy content involves within 1 year	N: [0, 1]	
X7 Policy Measures	X7:1 Legal Safeguards	Whether the policy involves legal safeguards	N: [0, 1]	[29, 34]
	X7:2 Interdepartmental coordination	Whether the policy involves coordination among government departments	N: [0, 1]	
	X7:3 Talent development	Whether the policy involves the cultivation or training of healthcare professionals	N: [0, 1]	
	X7:4 Performance assessment	Whether the policy includes assessment or evaluation mechanisms	N: [0, 1]	
	X7:5 Financial support	Whether the policy involves financial support	N: [0, 1]	
X8 Policy Evaluation	X8:1 Sufficient evidence base	Whether the policy is based on sufficient evidence or rationale	N: [0, 1]	[17, 27]
	X8:2 Clear objectives	Whether the policy has clearly defined objectives	N: [0, 1]	
	X8:3 Scientific program	Whether the policy program is scientific	N: [0, 1]	
	X8:4 Detailed planning	Whether the policy planning is detailed	N: [0, 1]	
X9 Policy Category	X9:1 Planning-type documents	Whether the policy is categorized as a law, plan, outline, regulation, normative standard, or strategy	1	[31]
	X9:2 Programmatic-type documents	Whether the policy is categorized as a program, detailed rule, guidance, or guidelines	0.8	
	X9:3 Measures-type documents	Whether the policy is categorized as measures, implementation measures, or administrative regulations	0.6	
	X9:4 Opinion-type documents	Whether the policy is categorized as opinions or decisions	0.4	
	X9:5 Notice-type documents	Whether the policy is categorized as a notice or announcement	0.2	

Notably, under the “Policy Category (X9)” variable, the secondary indicators are mutually exclusive. To ensure that this variable accurately captures differences in the strategic level and institutional attributes of policies, a separate reassignment rule is applied to X9. Specifically, the core content of the policy text serves as the basis for classification, with scoring conducted according to the criteria in Table 3. For instance, policy sample P9, entitled “Notice on Issuing the 13th Five-Year Plan for Deepening the Reform of the Medical and Healthcare System,” centers on a plan for deepening healthcare reform and is therefore classified as a “Planning-Type” policy, receiving the corresponding score.

To facilitate subsequent model calculations and visualization, the secondary indicator assignments were organized into a multi-input–output table, which serves as the basic data structure for subsequent PMC-index calculations and policy consistency evaluation, as shown in Table 4.

**Table 4:** Multi–input–output table

Primary Variable	X1	X2	X3	X4	X5	X6	X7	X8	X9
Secondary Variable	X1:1	X2:1	X3:1	X4:1	X5:1	X6:1	X7:1	X8:1	X9:1
	X1:2	X2:1	X3:2	X4:2	X5:2	X6:2	X7:2	X8:2	X9:2
	X1:3	X2:3	X3:3	X4:3	X5:3	X6:3	X7:3	X8:3	X9:3
	X1:4	X2:4	X3:4	X4:4		X6:4	X7:4	X8:4	X9:4
	X1:5	X2:5					X7:5		X9:5
	X1:6	X2:6							

#### Measurement of the PMC-index and policy grade classification

The PMC-index was calculated in four steps. First, the primary and secondary variables of each policy sample were placed into a multi-input-output table, as shown in Eq. (1). Second, binary values of [0, 1] were assigned to the secondary variables: a value of 1 when the policy text contained keywords or content consistent with the corresponding secondary variable, and 0 otherwise, as shown in Eq. (2). All policy texts were initially coded by the first author according to the predefined coding rules for each secondary variable. To formally assess coding reliability, independent blind double-coding was conducted by the corresponding author as a second coder on a stratified random sample of 8 of the 16 policies (50%), drawn proportionally from each policy stage. For the 36 binary variables under X1–X8 (288 paired judgments), Cohen’s Kappa coefficient was 0.86, indicating a high level of inter-rater agreement. For X9, a five-level ordered categorical variable, the Weighted Kappa coefficient was 1.00, indicating perfect agreement across the 8 sampled policies. All 14 discrepancies were resolved through structured discussion between the two coders, and the post-reconciliation codes were used in the final PMC-index calculations. Third, each primary variable score was calculated as the mean of its associated secondary variable scores, as shown in Eq. (3). Fourth, the PMC-index was calculated as the sum of all primary variable scores, as shown in Eq. (4), reflecting the consistency and overall quality of the policy text.

1 *X ∼ N[0,1]*

2 $$X = \left\{ {{X_R}:[0,1]} \right\}$$

3 $${X_i} = \left( {\sum\limits_{j = 1}^n {{{{X_{ij}}} \over {T({X_{ij}})}}} } \right)$$

4 $$PMC = \left[ \begin{gathered}{X_1}\left( {\sum\limits_{j = 1}^6 {\frac{{{X_{1j}}}}{6}} } \right) + {X_2}\left( {\sum\limits_{j = 1}^6 {\frac{{{X_{2j}}}}{6}} } \right) + {X_3}\left( {\sum\limits_{j = 1}^4 {\frac{{{X_{3j}}}}{4}} } \right) \hfill \\+ {X_4}\left( {\sum\limits_{j = 1}^4 {\frac{{{X_{4j}}}}{4}} } \right) + {X_5}\left( {\sum\limits_{j = 1}^3 {\frac{{{X_{5j}}}}{3}} } \right) + {X_6}\left( {\sum\limits_{j = 1}^4 {\frac{{{X_{6j}}}}{4}} } \right) \hfill \\+ {X_7}\left( {\sum\limits_{j = 1}^5 {\frac{{{X_{7j}}}}{5}} } \right) + {X_8}\left( {\sum\limits_{j = 1}^4 {\frac{{{X_{8j}}}}{4}} } \right) + {X_9} \hfill \\ \end{gathered} \right]$$

Here, *i = 1,2, … ,m* and *j = 1,2, … ,n* denote the indices of the primary and secondary variables, respectively. ${X_{ij}}$ represents the score of the $j{\rm{ - th}}$ secondary variable within the $i{\rm{ - th}}$ primary variable, while $T({X_{ij}})$ denotes the number of secondary variables within the $i{\rm{ - th}}$ primary variable. Based on the calculated PMC-index scores for each policy, the policy samples were classified into different quality grades. The corresponding evaluation criteria are presented in Table 5.

**Table 5:** Evaluation criteria for policies on the PMC-index

PMC-index	Policy Grade
8.00 ≤ PMC＜9.00	Excellent
6.00 ≤ PMC＜8.00	Good
4.00 ≤ PMC＜6.00	Acceptable
0.00 ≤ PMC＜4.00	Poor

#### Construction of the PMC-surface

The PMC-surface chart provides a visual representation of policy evaluation results in a multidimensional coordinate system, facilitating intuitive assessment and comparison of medical resource allocation policies. As shown in Eq. (5), the scores of the nine primary variables were transformed into a 3 × 3 matrix according to predetermined rules. Using the matrix elements as coordinates, nine evaluation points were plotted in three-dimensional space to generate the PMC-surface chart, with different colors indicating varying score levels. The convexity and concavity of each point and its surrounding neighborhood reflect the relative strengths and weaknesses of a policy across dimensions, providing an intuitive basis for identifying structural deficiencies.

5 $$PMC - Surface = \left[ {\matrix{ {{X_1}} & {{X_2}} & {{X_3}} \cr {{X_4}} & {{X_5}} & {{X_6}} \cr {{X_7}} & {{X_8}} & {{X_9}} \cr } } \right]$$

## Results

### Evaluation results for overall quality

Based on the PMC-index model and multiple-input-output tables constructed above, this study coded the secondary variables of the 16 policy samples according to the specific content of the policy texts. The scores of the primary variables were then calculated to derive the PMC-index and the corresponding policy grade for each policy. Results are presented in Table 6.

**Table 6:** The PMC-index sore and grade of policies

Policy	X1	X2	X3	X4	X5	X6	X7	X8	X9	PMC-index	Grade
P1	0.83	0.83	1.00	1.00	1.00	0.50	0.60	0.50	0.40	6.67	Good
P2	0.83	1.00	0.75	1.00	1.00	0.25	0.60	0.50	1.00	6.93	Good
P3	0.83	0.83	0.75	1.00	1.00	0.25	0.60	1.00	1.00	7.27	Good
P4	0.83	0.83	0.75	1.00	0.67	0.25	0.80	1.00	1.00	7.13	Good
P5	0.83	0.83	0.50	0.75	1.00	0.50	0.80	0.75	0.40	6.37	Good
P6	0.83	1.00	0.75	1.00	1.00	0.50	0.80	0.75	0.40	7.03	Good
P7	0.50	0.83	0.75	1.00	0.67	0.25	0.80	0.50	1.00	6.30	Good
P8	0.83	1.00	1.00	1.00	1.00	0.25	1.00	1.00	1.00	8.08	Excellent
P9	0.83	1.00	1.00	1.00	1.00	0.50	1.00	1.00	1.00	8.33	Excellent
P10	0.83	0.83	1.00	1.00	1.00	0.50	0.80	1.00	0.40	7.37	Good
P11	0.50	0.67	0.50	0.75	1.00	0.25	0.60	0.75	0.20	5.22	Acceptable
P12	0.50	0.67	0.50	1.00	1.00	0.25	0.80	0.75	0.20	5.67	Acceptable
P13	0.50	0.83	0.75	1.00	1.00	0.25	0.60	0.75	0.20	5.88	Acceptable
P14	0.67	0.67	1.00	1.00	1.00	0.50	1.00	0.50	0.40	6.73	Good
P15	0.50	0.67	0.50	1.00	0.67	0.25	0.60	0.75	0.20	5.13	Acceptable
P16	0.67	0.83	0.75	0.75	1.00	0.50	0.60	0.75	0.80	6.65	Good
Average	0.71	0.83	0.77	0.95	0.94	0.36	0.75	0.77	0.60	6.67	Good

Note: Minor discrepancies (within ±0.01) between the sum of primary variable scores and the reported PMC-index values may result from rounding during display formatting. All PMC-index calculations are based on the unrounded underlying values. This note applies to all subsequent tables reporting PMC-index values

Table 6 indicates that the average PMC-index of the 16 policy samples selected in this study is 6.67, corresponding to a “Good” overall level. Of these, two policies achieved an “Excellent” rating, ten received a “Good” rating, four were rated “Acceptable”, and none were classified as “Poor.” This suggests that China’s medical resource allocation policies exhibit a relatively high level of overall quality, with policy design demonstrating strong systemic coherence and coordination. These policies provide essential institutional support for optimizing medical resource allocation and ensuring rational use.

**Fig. 3:**
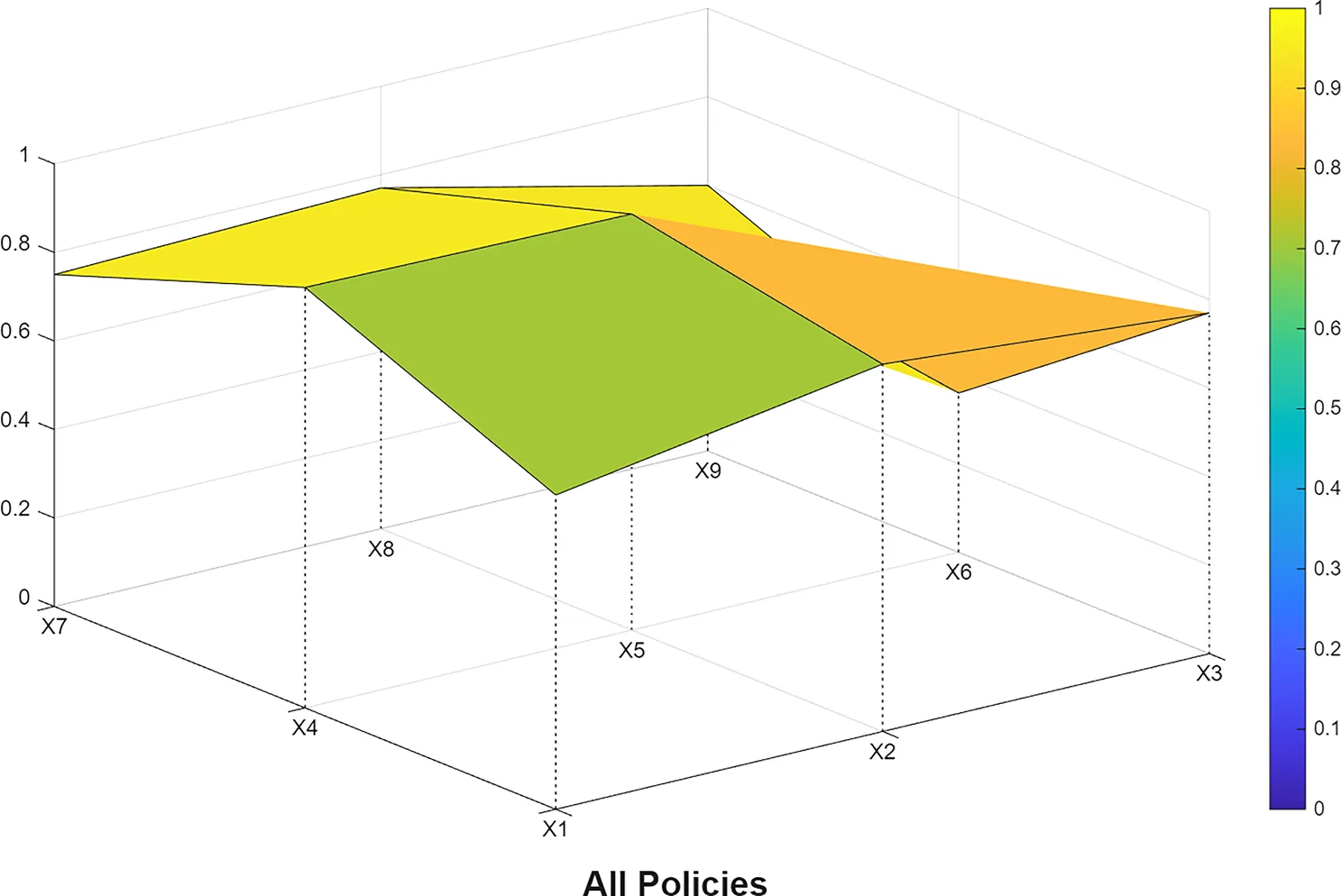
PMC-surface chart for all policies

**Fig. 4:**
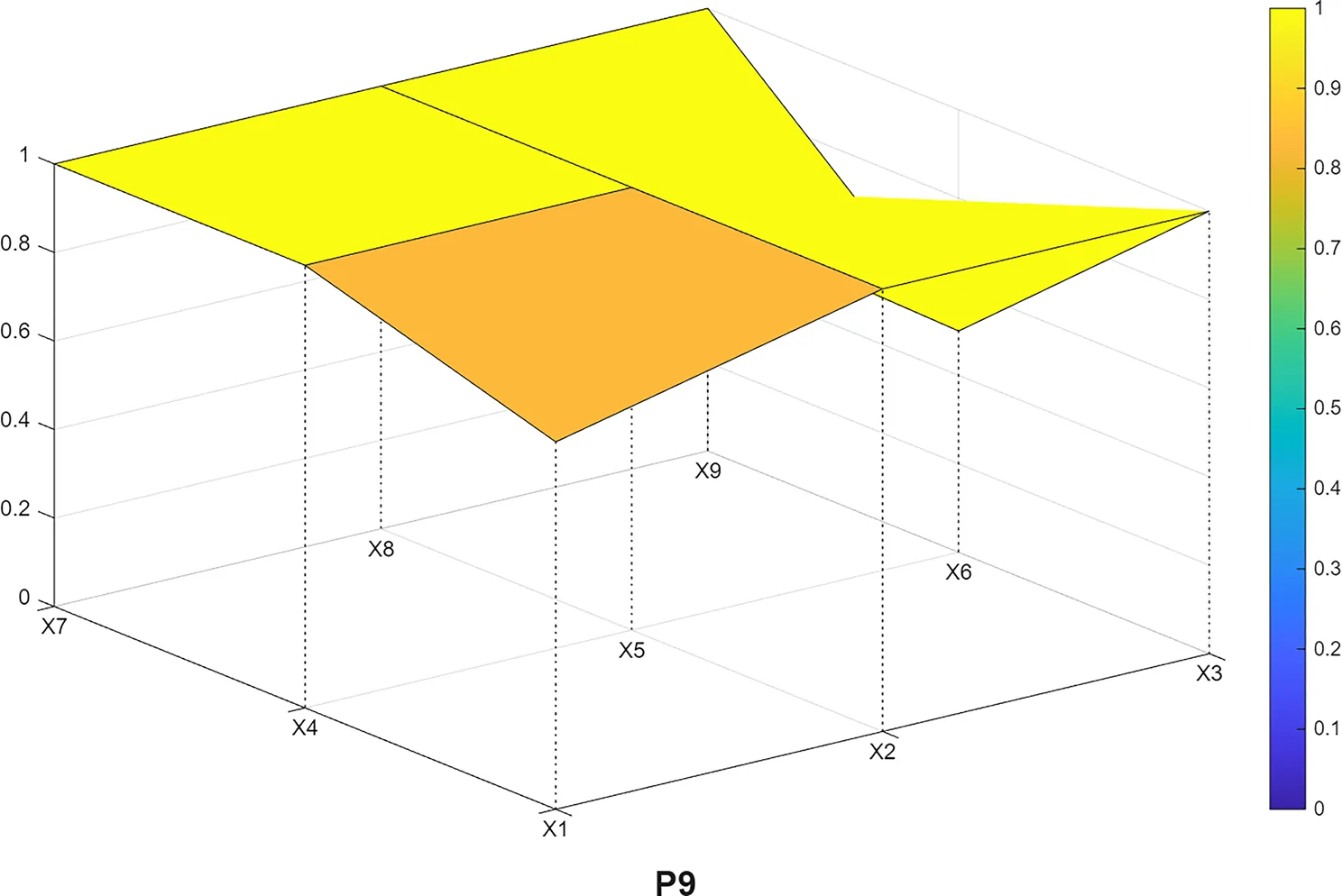
PMC-surface chart for P9 (highest-scoring policy)

**Fig. 5:**
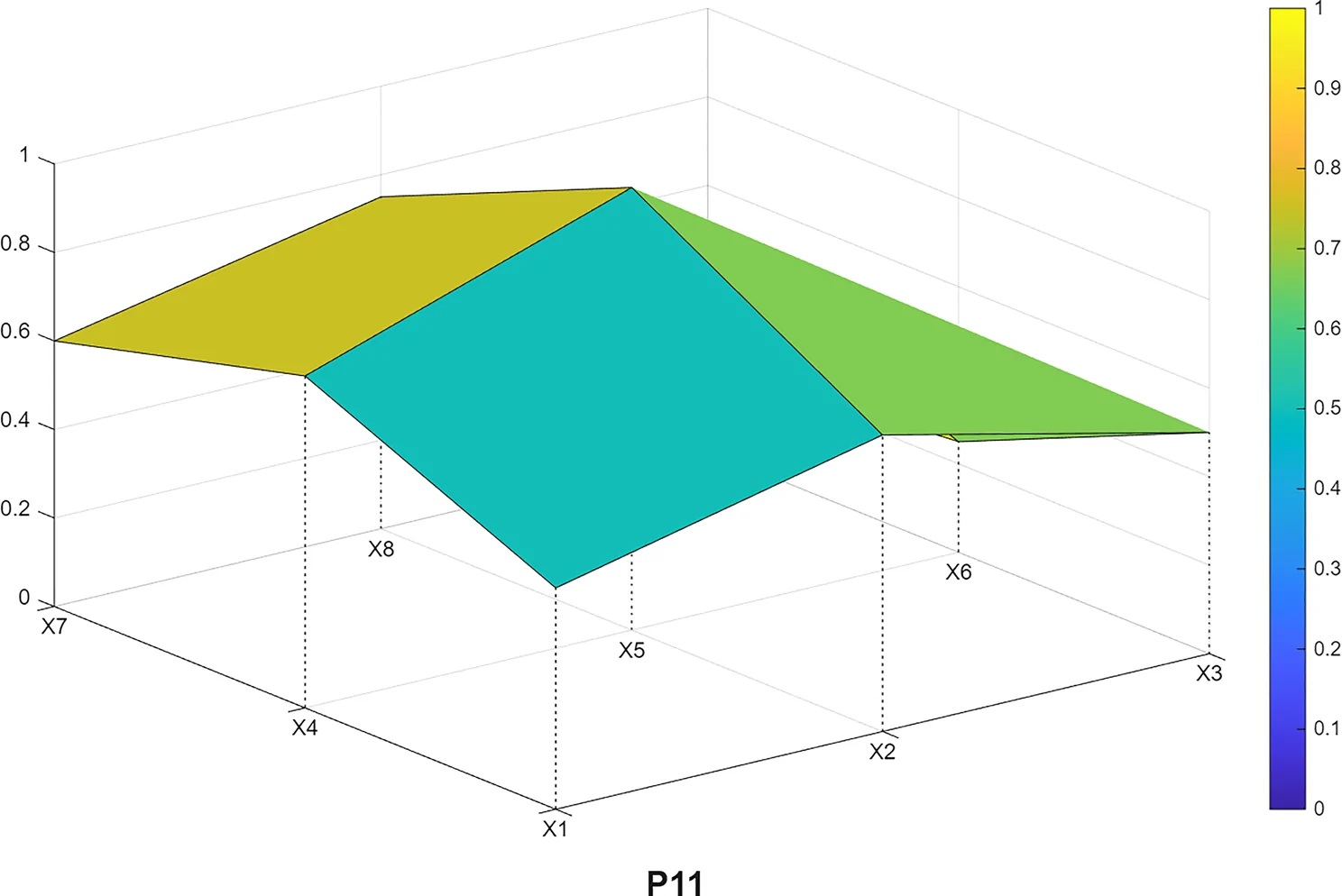
PMC-surface chart for P11 (lowest-scoring policy)

To further illustrate structural differences across policy dimensions, MATLAB R2025a was used to generate PMC-surface charts for all policy samples. For comparison, this study selected the highest-scoring policy (P9) and the lowest-scoring policy (P11) (see Figs. 3, 4 and 5). In these PMC-surface charts, convex regions indicate the policies’ relative strengths, whereas concave areas reflect their relative weaknesses.

Based on Table 6 and Figs. 3, 4 and 5, the primary variable scores show that X4 (Policy Target) achieved the highest value. This indicates comprehensive coverage of its target groups and effectively balancing of the interests of diverse stakeholders, including medical institutions, healthcare professionals, and patients. Such a pattern reflects a high degree of fairness and inclusivity in the policy formulation. X5 (Policy Instruments) attained an average score of 0.94, showing that the policy instrument system is comprehensive and well structured. It incorporates both direct instruments (e.g., increasing resource supply) and indirect instruments (e.g., guiding demand and optimizing the policy environment), generating synergistic effects that improve policy implementation outcomes. X2 (Policy Content) scored 0.83, indicating relatively high comprehensiveness. China’s medical resource allocation policies generally address key areas such as optimizing resource distribution, strengthening primary healthcare service capacity, and promoting collaboration among healthcare institutions. These measures help alleviate imbalances in both the total volume and structural distribution of medical resources. However, provisions related to public hospitals scale management and differentiated health insurance payment mechanisms remain relatively insufficient, suggesting a need for further refinement to curb the disorderly expansion of large public hospitals to better leverage health insurance payment in guiding patient flows.

X3 (Policy Objective) and X8 (Policy Evaluation) both scored 0.77, indicating above-average levels. The overall goal orientation of medical resource allocation policies is therefore relatively clear, reflecting balanced consideration of multidimensional objectives including equity, efficiency, institutional reform, and universal health coverage. However, although some policies articulate explicit objectives, their underlying rationale and detailed implementation plans remain insufficient. Further refinement through supplementary implementation rules and improved execution procedures is needed to enhance policy operability and scientific rigor.

X1 (Policy Nature, 0.71) and X7 (Policy Measures, 0.75) fall within the medium range, indicating that China’s medical resource allocation policies contain certain guiding principles and implementation arrangements but leave substantial room for improvement. For X1(Policy Nature), policies tend to emphasize descriptive and guidance-oriented provisions, while predictive and recommendatory content remains limited. This suggests a lack of foresight in anticipating future trends and designing forward-looking frameworks for medical resource allocation. For X7 (Policy Measures), although interdepartmental coordination and fiscal support are commonly included, legal safeguards and performance evaluation mechanisms remain relatively weak, leaving a complete closed-loop system linking goal setting, implementation, and outcome assessment yet to be established.

X6 (Policy Timeliness) and X9 (Policy Category) scored relatively low, at 0.36 and 0.60, respectively. The low X6 score indicates that some policies have relatively short validity periods and lack medium- to long-term planning guidance, resulting in insufficient policy continuity and stability that hinder the establishment of institutionalized and normalized policy arrangements. The low X9 score reflects the predominance of guidance-based documents in the policy sample, such as notices and opinions, while higher-level policy instruments, including laws and strategic plans, remain relatively scarce. Consequently, the institutional authority and binding force of medical resource allocation policies require further strengthening.

Overall, China’s medical resource allocation policies demonstrate relatively good quality, yet continuous improvement is needed, particularly in enhancing policy foresight and binding force, strengthening high-level institutional design, and developing more detailed and operational supporting rules.

### Evaluation results for policies of different quality grades

**Fig. 6:**
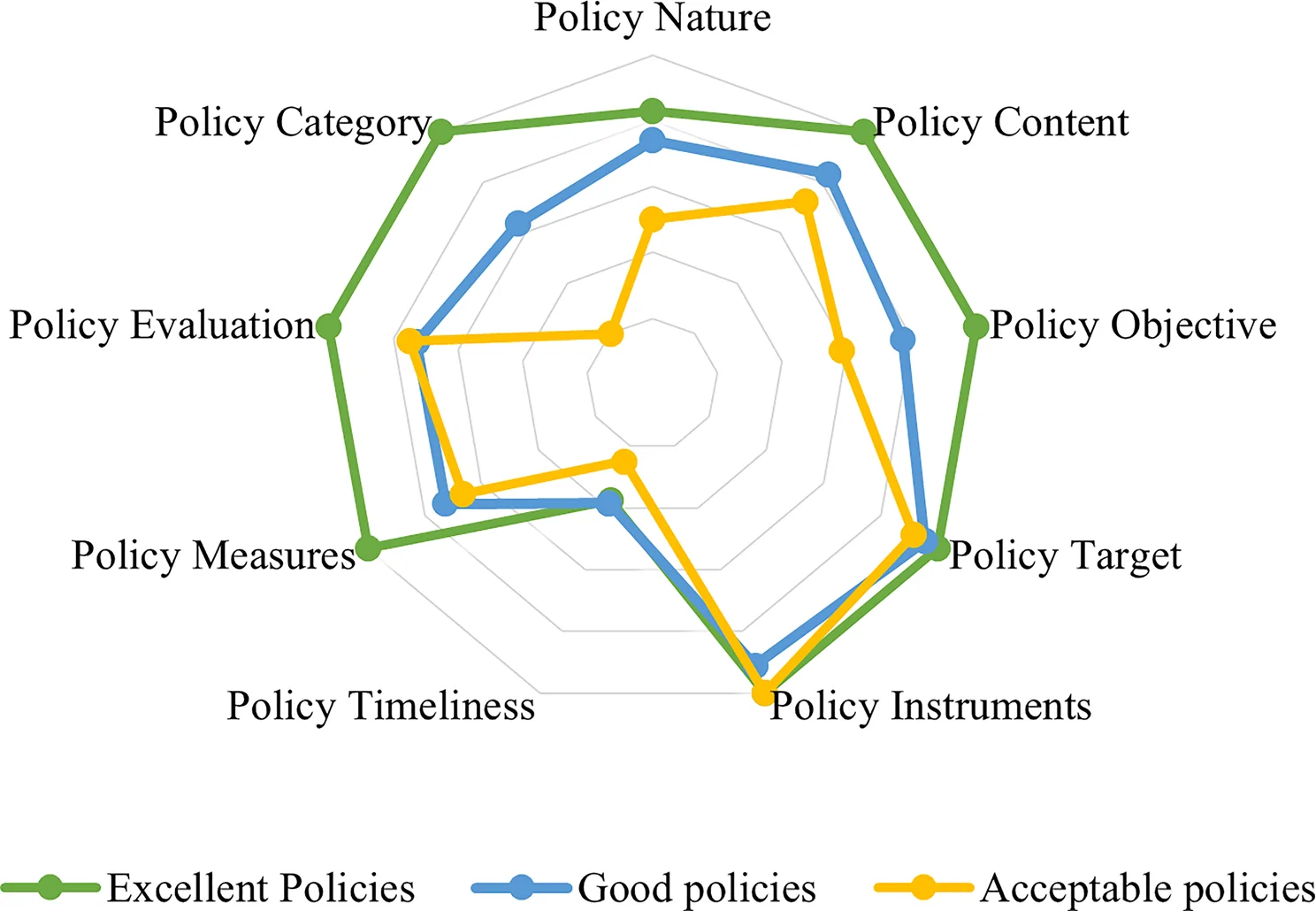
Comparison of policies with different quality grades

To visualize structural differences across evaluation dimensions among policies of different quality grades, this study first presents radar charts for “Excellent”, “Good”, and “Acceptable” policies (see Fig. 6). The figure reveals that excellent policies consistently score near the outer ring across multiple dimensions, including X2 (Policy Content), X3 (Policy Objective), X4 (Policy Target), X5 (Policy Instruments), X7 (Policy Measures), and X8 (Policy Evaluation), exhibiting the most complete policy profile. Good policies exhibit slightly lower overall scores but maintain a broadly similar structural shape, indicating relatively high internal consistency. In contrast, acceptable policies display pronounced contraction in several dimensions, such as X1 (Policy Nature), X2 (Policy Content), X3 (Policy Objective), and X9 (Policy Category), suggesting relatively low institutional completeness.

**Fig. 7:**
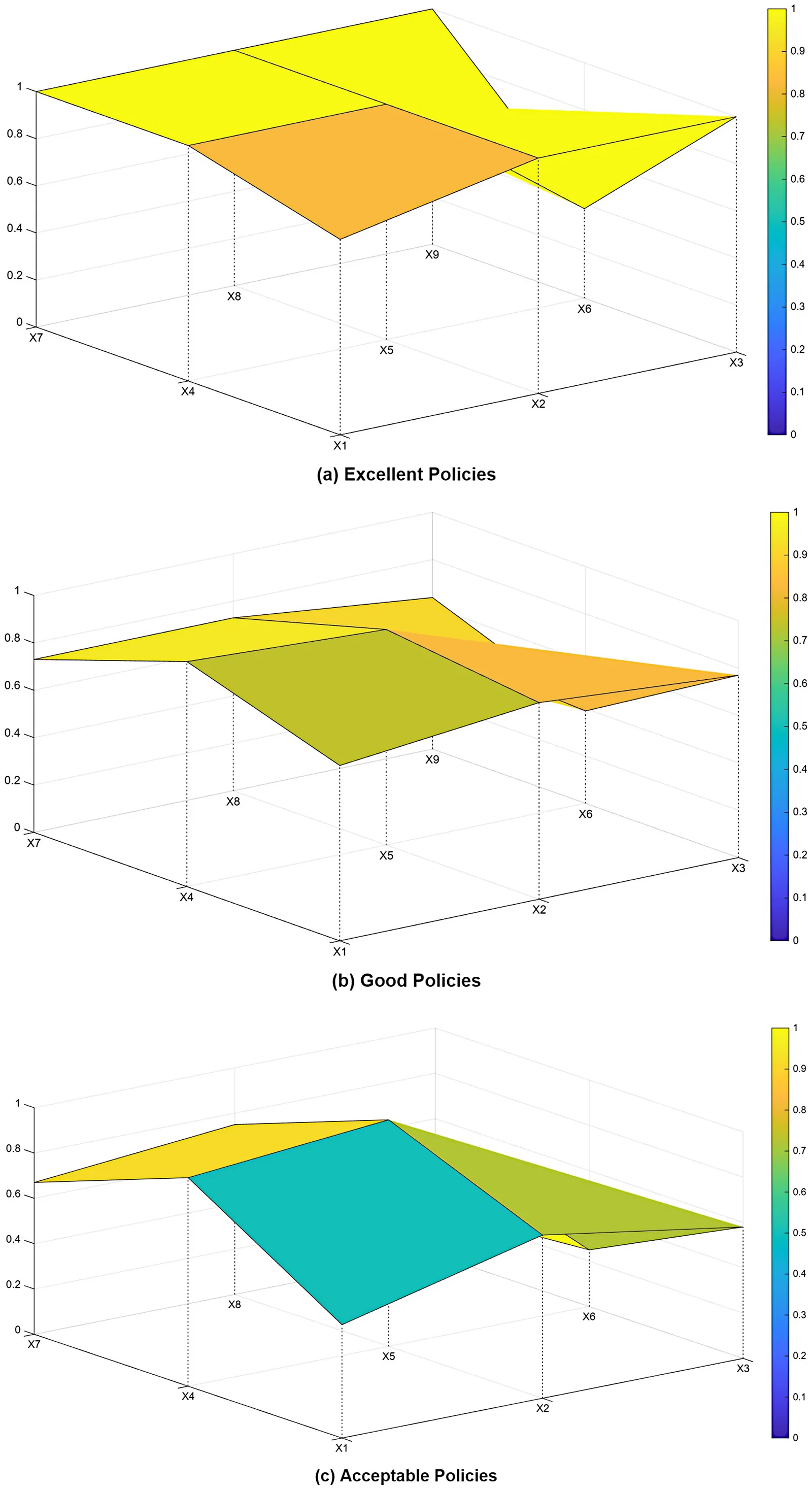
PMC-surface charts for policies with different quality grades

To reveal specific differences and structural characteristics among policies within the same quality grade, MATLAB R2025a was used to generate PMC-surface charts for excellent, good, and acceptable policies (as shown in Fig. 7). A further comparative analysis of the PMC-index indicates that policy performance on each primary variable varies significantly across quality grades.

1. **Excellent Policies.** Two policies are classified as excellent: P8 and P9. Across multiple dimensions, including X2 (Policy Content), X3 (Policy Objective), X4 (Policy Target), X5 (Policy Instruments), X7 (Policy Measures), X8 (Policy Evaluation), and X9 (Policy Category), the corresponding indicators either approach or achieve full marks. These policies exhibit high comprehensiveness in content coverage, objective formulation, implementation instruments, and supporting systems, generally representing well-coordinated top-level design documents. They not only clearly articulate strategic priorities, such as optimizing medical resource allocation, enhancing primary healthcare service capacity, and promoting institutional reform, but also strengthen their authority and binding force through legal or planning-based frameworks, thus playing a foundational and guiding role within the overall policy system. However, that the average score for Policy Timeliness (X6) is only 0.38, which is consistent with the overall sample average, indicating substantial room for improvement in medium- to long-term policy design and dynamic adjustment mechanisms.
2. **Good Policies.** Ten policies are rated as good: P1, P2, P3, P4, P5, P6, P7, P10, P14, and P16. Their scores across primary indicators generally approximate the sample average, demonstrating overall robustness despite minor internal imbalances. These policies perform particularly well on Policy Target (X4 = 0.95), Policy Instruments (X5 = 0.91), Policy Content (X2 = 0.83), and Policy Objective (X3 = 0.77). This indicates that these policies have a relatively sound institutional design for addressing imbalances in medical resource allocation while balancing equity and efficiency, and that they provide effective of key stakeholders, including healthcare administrative departments and healthcare institutions. For Policy Measures (X7 = 0.73), interdepartmental coordination and fiscal support arrangements are relatively well established, complemented by clear implementation plans and detailed execution strategies (Policy Evaluation, X8 = 0.73). However, compared with excellent policies, good policies still exhibit minor deficiencies in Policy Nature (X1) and Policy Category (X9), some remain primarily departmental rules or normative documents, reflecting relatively weaker authority and sustainability.

Overall, good policies constitute the main component of China’s medical resource allocation policy system, serving as the primary driving force behind related reforms. Nevertheless, there remains scope for advancement toward the excellent category through measures such as elevating policy status and strengthening evaluation and feedback mechanisms.

(3) **Acceptable Policies.** Four policies are classified as acceptable: P11, P12, P13, and P15. This category scores significantly lower than the preceding two on most primary indicators, particularly Policy Nature (X1 = 0.50), Policy Content (X2 = 0.72), and Policy Objective (X3 = 0.58), reflecting relative deficiencies in foresight, systematic design, and objective clarity. The policy texts often remain at the level of general principles or simple restate higher-level documents, lacking substantive responses to core issues in medical resource allocation. Their Policy Category (X9) of only 0.20 indicates that these policies are primarily notices, opinions, or general work arrangements, with limited regulatory authority and binding force. The Policy Timeliness (X6) score of 0.25 demonstrates a strong focus on short-term or annual tasks, with relatively weaker orientation toward medium- to long-term planning.

However, these policies still perform relatively well in Policy Instruments (X5 = 1.00) and Policy Evaluation (X8 = 0.75), demonstrating a solid foundation in specific implementation measures and basic planning arrangements, functioning primarily as implementation-oriented or operational policies. Future efforts should further enrich policy objectives and content design while establishing closer alignment with higher-level policies such as laws and master plans. Overall, substantial scope for improvement remains in the quality of acceptable policies.

### Evaluation results for policies at different stages

To systematically examine the evolutionary trajectory of China’s medical resource allocation policy reforms, this study divides the policy sample into three developmental stages based on key temporal milestones: the Exploration Stage (2009–2015), the Deepening Stage (2016–2020), and the Focus Stage (2021–2025). Each stage exhibits distinct policy orientations and developmental characteristics.

The first stage began with the 2009 “Opinions on Deepening the Reform of the Medical and Health System,” which marked the comprehensive launch of a new round of medical and health system reforms and established the top-level institutional framework for medical resource allocation reform. Policy practice during this stage focused primarily on macro-level institutional design and exploring pathways for medical resource allocation.

The second stage, coinciding with the 13th Five-Year Plan (2016–2020), marked a phase of institutional deepening for medical resource allocation reforms. Its defining feature was the nationwide systematic advancement of collaborative resource allocation mechanisms, exemplified by medical consortiums development.

The third stage, beginning with the 14th Five-Year Plan (2021–2025), witnessed a pronounced shift toward more refined and decentralized policy orientations. Policy focus progressively emphasized systematic enhancement of primary healthcare service capacity. Through measures such as improving the hierarchical medical service system and promoting the downward allocation of high-quality medical resources, efforts aimed to establish a multi-tiered and equitable structure for medical resource allocation.

Based on this temporal segmentation, this study further conducts a heterogeneous analysis of policy characteristics across the three stages. Table 7 presents the PMC-index results for each stage, with six, five, and five policies enacted during the Exploration, Deepening, and Focus stages, respectively.

**Table 7:** The PMC-index score for different stages

Stages	X1	X2	X3	X4	X5	X6	X7	X8	X9	PMC-index
Exploration Stage (2009–2015)	0.83	0.89	0.75	0.96	0.95	0.38	0.70	0.75	0.70	6.90
Deepening Stage (2016–2020)	0.70	0.87	0.85	0.95	0.93	0.35	0.84	0.85	0.72	7.06
Focus Stage (2021–2025)	0.57	0.73	0.70	0.95	0.93	0.35	0.72	0.70	0.36	6.02

Table 7 reveals distinct evolutionary characteristics across stages. Overall, the PMC-index stood at 6.90 during the Exploration Stage (2009–2015), increased to 7.06 in the Deepening Stage (2016–2020), and then declined to 6.02 in the Focus Stage (2021–2025), forming an inverted-U trajectory in policy quality. Examining the primary indicators reveals consistently high and stable scores across all three stages for Policy Target (X4, consistently above 0.95) and Policy Instruments (X5, consistently above 0.93), indicating broad coverage of key stakeholders and employed a combination of supply-side, demand-side, and environmental instruments. In contrast, Policy Timeliness (X6) scored relatively low across all three stages (approximately 0.35–0.38), reflecting an overall lack of institutional arrangements for medium- to long-term planning, which remained a consistent weakness in medical resource allocation policies across all periods.

To assess the robustness of the inverted-U temporal trajectory and disentangle potential document-format effects from genuine changes in substantive policy content, this study conducted a sensitivity analysis under three alternative specifications, recalculating cross-stage scores after: (1) excluding X9 (Policy Category), (2) excluding both X6 and X9, and (3) computing the substantive content achievement as the mean of the five substantive content variables (X2, X3, X5, X7, X8). This third specification captures a broader substantive policy orientation while remaining less directly influenced by document format. Results are reported in Table 8.

**Table 8:** Sensitivity analysis of cross-stage PMC-index scores under alternative specifications

Stages	Full PMC-index (9 variables)	Excluding X9 (8 variables)	Excluding X6 and X9 (7 variables)	Substantive content avg. (X2, X3, X5, X7, X8)
Exploration Stage	6.90	6.20	5.82	0.81
Deepening Stage	7.06	6.34	5.99	0.87
Focus Stage	6.02	5.66	5.31	0.76

Note: The original PMC-index is based on nine dimensions (X1–X9). The adjusted scores excluding X9, and excluding both X6 and X9, are based on eight and seven dimensions, respectively, and are therefore used to test the robustness of cross-stage comparisons rather than for direct absolute comparison with the original PMC-index. The substantive content average is the unweighted mean of X2, X3, X5, X7, and X8

The inverted-U trajectory remains robust across all four specifications, with the Deepening Stage consistently highest and the Focus Stage lowest. After excluding X9, the gap between the Deepening Stage and the Focus Stage narrowed, suggesting that part of the apparent decline in the original PMC-index was associated with the framework’s lower scoring of execution-level policy documents. However, the Focus Stage remained the lowest-scoring period across all alternative specifications. Moreover, the substantive content average also declined from 0.87 in the Deepening Stage to 0.76 in the Focus Stage, indicating that the observed decline cannot be explained solely by document format but also reflects a moderate weakening in substantive policy content.

**Fig. 8:**
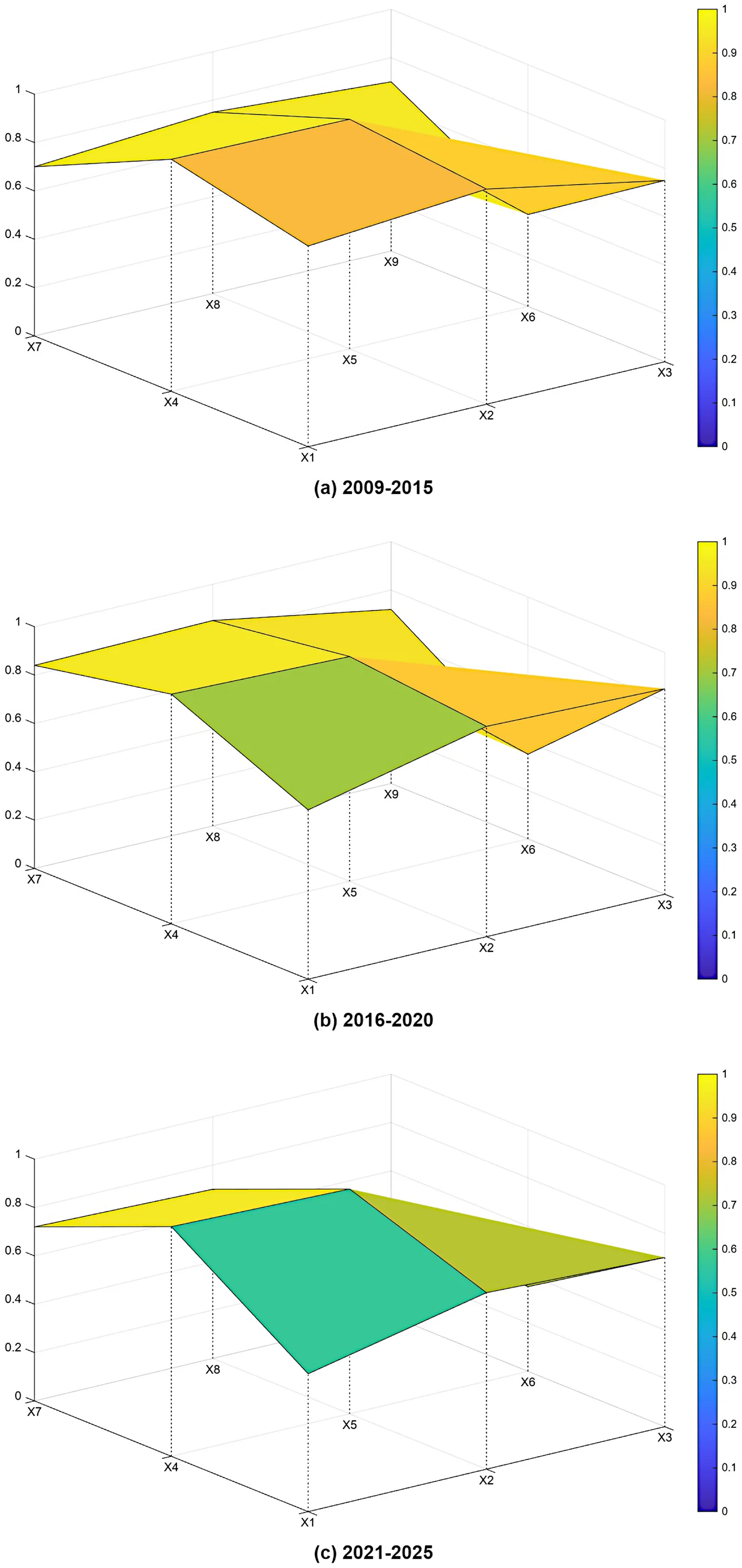
PMC-surface charts for policies at different stages

1. **Exploration Stage (2009–2015).** As shown in Table 7 and Fig. 8(a), the average PMC-index for policies during the Exploration Stage was 6.90, indicating relatively high overall quality. Amid the full-scale launch of the new healthcare reform, medical resource allocation policies initially demonstrated a relatively strong textual standardization and institutional design capacity.

Among the primary indicators, Policy Nature (X1 = 0.83), Policy Content (X2 = 0.89), and Policy Instruments (X5 = 0.95) scored relatively high, indicating that policies in this stage emphasized top-level design and institutional architecture. They were comprehensive in value orientation and content structure, establishing a foundational institutional framework for subsequent reform. In contrast, Policy Objective (X3 = 0.75), Policy Measures (X7 = 0.70), and Policy Evaluation (X8 = 0.75), although showing some institutional foundation, remained relatively weak in interdepartmental coordination, performance assessment mechanisms, and detailed implementation planning. The low Policy Timeliness score (X6 = 0.38) further reflected the underdevelopment of medium- to long-term institutional arrangements.

Overall, this stage was characterized by the establishment of a top-level institutional framework, with execution and implementation mechanisms still at an early exploratory phase.

(2) **Deepening Stage (2016–2020).** As shown in Table 7 and Fig. 8(b), the average PMC-index for policies during the Deepening Stage rose to 7.06, representing the highest overall quality among the three stages. Under the strategic coordination of the 13th Five-Year Plan, medical resource allocation policies entered a phase of institutional systematization and mechanism innovation.

In this stage, Policy Objective (X3 = 0.85), Policy Measures (X7 = 0.84), and Policy Evaluation (X8 = 0.85) reached their highest values across the three stages, reflecting a stronger focus on improving efficiency, advancing systemic reform, and strengthening interdepartmental coordination, performance assessment, financial support, and the rigor and detailed nature of implementation plans. Policy Content (X2 = 0.87) and Policy Target (X4 = 0.95) also remained at high levels, indicating that the nationwide promotion of coordinated mechanisms, such as medical consortia, advanced in a more systematic manner, with policies showing greater balance in targeting both key areas and critical stakeholders. Policy Instruments (X5 = 0.93) continued to reflect a hybrid deployment of supply-side, demand-side, and environmental instruments, underscoring the diversity and coordination of the policy toolkit.

Notably, however, Policy Nature (X1 = 0.70) declined compared with the Exploration Stage, and Policy Timeliness (X6 = 0.35) remained relatively low, implying that although policy execution and supporting mechanisms became more mature, room remained for improvement in predictive design, advisory components, and medium- to long-term strategic planning.

(3) **Focus Stage (2021–2025).** As shown in Table 7 and Fig. 8(c), the average PMC-index of policies during the Focus Stage declined to 6.02, lower than the previous two stages. This reflects a slight decrease in the overall textual quality of medical resource allocation policies as reform priorities shifted toward primary-level implementation and task-specific advancement. To some extent, this pattern also reflects a stage-specific shift in the institutional logic of healthcare reform: as the basic policy framework had already been largely established in earlier stages, policy development in this period placed greater emphasis on grassroots implementation, task-specific coordination, and the consolidation of existing reform arrangements.

Regarding indicator distribution, Policy Content (X2 = 0.73), Policy Objective (X3 = 0.70), Policy Measures (X7 = 0.72), and Policy Evaluation (X8 = 0.70) all declined compared with earlier periods, indicating that policies at this stage became relatively weaker in content systematization, clarity of objective-setting, and the completeness of supporting execution mechanisms. A particularly notable decline was observed in Policy Category (X9 = 0.36), suggesting that a larger proportion of documents in the sample were operational in nature, such as notices, opinions, and annual work plans, while programmatic documents such as high-level plans and strategic outlines became less frequent. This structural shift helps explain interpreting the lower PMC-index score. As confirmed by the sensitivity analysis, the predominance of execution-level policy documents partly contributed to the lower score observed in the Focus Stage under the current evaluation framework, although it did not fully account for the decline. Meanwhile, Policy Timeliness (X6 = 0.35) remained relatively low, indicating that medium- to long-term institutional arrangements were still insufficient. Nevertheless, Policy Target (X4 = 0.95) and Policy Instruments (X5 = 0.93) remained at high levels, suggesting a relatively stable pattern in target group coverage and the combination of policy instruments.

The Focus Stage is therefore characterized by strong implementation but weak frameworks and long-term vision, revealing a shift in reform priorities from institutional design and mechanism innovation toward grassroots-level execution and task-specific implementation. This also implies that future policies, while maintaining this implementation-oriented approach, should appropriately strengthen policy hierarchy, forward-looking design, and medium- to long-term strategic planning.

Overall, the evolution of medical resource allocation policies has followed a trajectory from macro-level top-down design during the Exploration Stage, to institutional improvement and mechanism innovation in the Deepening Stage, and finally to grassroots-oriented implementation and task-specific refinement in the Focus Stage. Although the most recent stage showed some decline in policy hierarchy and systemic coherence, it also reflects a realistic shift in reform logic, from overall institutional construction toward strengthening grassroots implementation and execution effectiveness. This transformation provides valuable insights for achieving a better balance between improving policy quality and enhancing policy enforceability in the future.

## Conclusions and policy implications

### Conclusions

This study examines national-level policies on medical resource allocation. By integrating text-mining techniques with the PMC-index model and drawing on a systematic review of the existing literature, it constructs a comprehensive policy evaluation framework comprising nine primary variables and 41 secondary variables. A total of 16 policy documents were selected for quantitative evaluation and comparative analysis. The main conclusions are as follows:

First, China’s national-level medical resource allocation policies demonstrate relatively high overall quality. The 16 selected policies yielded an average PMC-index score of 6.67, placing them within the “Good” range. Among these, two policies were rated “Excellent,” ten as “Good,” and four “Acceptable,” with none classified “Poor”. This suggests that current national policies on medical resource allocation are generally well structured and scientifically grounded, providing institutional support for the optimized distribution and rational utilization of medical resources.

Second, policies of different quality grades show distinct emphases in functional orientation and institutional design while exhibiting collective complementarity. Comparative analysis reveals that “Excellent” policies score near full marks across multiple dimensions, characterized by broad content coverage, comprehensive institutional frameworks, and a high policy hierarchy, primarily serving top-level design and overarching strategic functions. “Good” policies, which form the majority, perform stably on Policy Content (X2), Policy Objective (X3), and Policy Instruments (X5), with strong specificity and operability, functioning as the main drivers of reform implementation. “Acceptable” policies are comparatively weaker in policy nature, content depth, and clarity of objectives, often taking the form of notices, opinions, or annual work plans. Nonetheless, they still provide a foundation for implementation measures and action plans. Overall, these different categories of policies complement each other in functional positioning, contributing to the integrity and coordination of the medical resource allocation policy system.

Third, structural weaknesses exist in the categorical dimension of medical resource allocation policies. Analysis of the primary variables reveals a certain degree of convergence across China’s policies in this field, with most performing strongly on Policy Target, Policy Instruments, and Policy Content. This suggests that national-level policies effectively cover key stakeholders, including health administrative departments, healthcare institutions, and patients, while employing a diverse mix of policy instruments. However, the relatively low scores for X6 (Policy Timeliness) and X9 (Policy Category) indicate a lack of medium- to long-term planning and an insufficient supply of high-level legal, strategic, and programmatic documents, which constitute common institutional weaknesses in the current policy structure. Furthermore, temporal analysis of PMC-index scores shows an upward trend from the Exploration Stage (2009–2015) to the Deepening Stage (2016–2020), followed by a decline during the Focus Stage (2021–2025). This pattern reflects a shift in policy emphasis from framework construction to grassroots implementation and task-level advancement, which has, to some extent, weakened systemic coherence and policy hierarchy.

Fourth, existing medical resource allocation policies require further improvement in forward-looking design and the establishment of long-term mechanisms. The PMC-index results indicate that, although current policies have established a relatively comprehensive institutional framework at the textual level, several areas still leave room for improvement: (1) the predictive and anticipatory capacity of these policies remains insufficient, with limited consideration of future trends in healthcare service demand and resource distribution; (2) their timeliness and orientation toward medium- to long-term planning remain weak, as many policies have short implementation cycles and lack timeframes aligned with long-term health strategies; and (3) vertical coordination between high-level laws and strategic plans, on the one hand, and lower-tier operational documents, on the other, remains inadequate, and a closed-loop governance system spanning top-level design, grassroots implementation, and feedback-based evaluation has yet to be fully established.

### Policy implications

Building on the quantitative evaluation above and the structural weaknesses identified, this study proposes the following policy implications, aimed at providing concrete policy instruments and implementation pathways for improving China’s medical resource allocation policy system.

First, policy hierarchy should be strengthened to enhance institutional authority and long-term stability. The relatively low score of Policy Category (X9) suggests that the current policy system relies heavily on lower-level documents, while higher-level legal, strategic, and programmatic documents remain insufficient. China should therefore strengthen national strategic planning and medium- to long-term policy design, while improving coordination between top-level policy frameworks and lower-level implementation rules. Provincial and local governments should translate national objectives into region-specific resource allocation plans based on demographic trends, disease burden, and service capacity. International experience also suggests that effective medical resource allocation depends on clear hierarchical planning and strong coordination between national strategy and local implementation.

Second, forward-looking design and long-term planning mechanisms should be improved. The persistently low score of Policy Timeliness (X6) indicates that current policies remain more focused on short-term tasks than on sustained institutional planning. Future policymaking should establish a governance framework that links long-term strategic goals, medium-term plans, and annual tasks. Major policies should set clear five-year objectives, phased targets, and regular review procedures. Policy design should also pay closer attention to major structural changes, including population aging, shifts in disease patterns, urban-rural demographic changes, and advances in digital health, and incorporate these factors into resource demand forecasting and capacity planning to improve policy adaptability.

Third, policy instruments for controlling the scale of large hospitals and guiding differentiated resource allocation should be refined. The analysis of secondary indicators shows that policy content related to public hospital scale management and differentiated medical insurance payment remains relatively weak. Future reforms should adopt a more targeted policy mix combining total volume control, structural adjustment, and grassroots-oriented resource guidance. Specifically, stricter regulation of bed expansion and high-value equipment allocation in large public hospitals should be introduced, while new high-quality resources should be directed more toward primary care institutions, county-level healthcare systems, and underdeveloped areas. Differentiated medical insurance payment policies should also be further improved to better support tiered diagnosis and treatment. For example, reimbursement and payment incentives can be aligned more closely with first-contact care at the grassroots level, particularly for common and chronic diseases. Rural areas, remote regions, and vulnerable populations should also receive more targeted support through fiscal transfers, workforce policies, and digital health infrastructure.

Fourth, a multi-level collaborative governance mechanism should be established to enhance systemic coordination and policy resilience. The stage heterogeneity analysis shows that, although recent policies have paid greater attention to grassroots implementation, their systemic coherence, policy hierarchy, and forward-looking design have weakened to some extent. Future policy development should therefore strengthen cross-level coordination, information sharing, and feedback adjustment. A collaborative governance framework should be established in which the central government focuses on strategic goals and regulatory standards, provincial authorities on regional coordination, and local governments, medical institutions, and medical insurance agencies on implementation and policy feedback. Performance evaluation and cross-departmental coordination should also be strengthened to gradually establish a closed-loop governance system covering policy formulation, implementation, monitoring, feedback, and adjustment. Through these efforts, China can further improve the coherence, adaptability, and long-term effectiveness of its medical resource allocation policy system.

### Limitations and future research

Although this study provides valuable insights into the quantitative evaluation of medical resource allocation policies in China, it has several limitations.

First, this study focuses on national-level formal policy documents and therefore does not cover all local or implementation-oriented texts related to medical resource allocation. Although more operational and explicit screening criteria were applied to improve comparability and transparency, some degree of sample selection constraint may remain. Nevertheless, the final sample was intended to capture the major national policy framework in this field. Future research could incorporate policy documents from different administrative levels to examine how policy priorities and instruments vary across central and local governments.

Second, although double-coding was conducted on 50% of the sample with high inter-rater reliability (Cohen’s Kappa = 0.86 for the 36 binary variables under X1–X8; Weighted Kappa = 1.00 for X9), the remaining 50% was coded by the first author only. Future research could further enhance robustness by extending double-coding to the full sample and by introducing multiple independent coders to assess reliability across diverse research backgrounds.

Third, the PMC-index model used in this study has certain methodological constraints. It may implicitly favor broad, strategic, and planning-oriented policy documents, while assigning relatively lower scores to shorter-term or execution-oriented documents. Although the sensitivity analysis confirmed that the Focus Stage remained the lowest-scoring period after adjusting for document-format-sensitive variables, the observed decline was nonetheless partly associated with this structural feature of the evaluation framework. In addition, the model assumes equal weights across evaluation dimensions, which may not fully reflect their relative importance in actual policymaking. Future research could further optimize the PMC-index model by introducing differentiated weighting methods, such as the Analytic Hierarchy Process or entropy weighting, and by integrating it with other analytical approaches, such as machine learning methods, to improve the accuracy and robustness of policy evaluation.

## Electronic supplementary material

Below is the link to the electronic supplementary material.

Supplementary Material 1

## Data Availability

Data is available from the corresponding author on request.
