# Supplementary material for: Quantitative evaluation of the medical resource allocation policy in China: a PMC-index model approach
Source: Cost Eff Resour Alloc. 2026 Jun 25;24:113. doi: 10.1186/s12962-026-00788-z (PMC13560427; doi:10.1186/s12962-026-00788-z)
Supplement: Supplementary file 1 [file 12962_2026_788_MOESM1_ESM.docx]

**Supplementary Table S1 Excluded policy documents and reasons for exclusion**

| **No.** | **Policy Title** | **Issuing Agency** | **Reason for exclusion** | **Year** |
| --- | --- | --- | --- | --- |
| 1 | Letter on Entrusting the Pilot Work of Optimizing Service Procedures to Improve the Efficiency of Medical Resource Utilization | General Office of the Ministry of Health (abolished) | Work letter format; incomplete policy text structure for PMC-index coding | 2009 |
| 2 | Notice on Issuing the Arrangements for the Pilot Reform of Public Hospitals in 2011 | General Office of the State Council | Mainly focused on public hospital reform; limited substantive content on medical resource allocation | 2011 |
| 3 | Notice on Issuing the Development Plan for the Service Sector during the Twelfth Five-Year Plan Period | the State Council | Mainly focused on service-sector development; medical resource allocation only briefly mentioned | 2012 |
| 4 | Several Opinions on Accelerating the Development of Commercial Health Insurance | General Office of the State Council | Mainly focused on commercial health insurance; medical resource allocation only incidentally mentioned | 2014 |
| 5 | Report on the Progress of Deepening the Reform of the Medical and Health System | the State Council | Progress report rather than a substantive policy text; medical resource allocation only briefly mentioned | 2015 |
| 6 | Guiding Opinions on the Pilot Reform of Urban Public Hospitals | General Office of the State Council | Mainly focused on urban public hospital reform; limited direct relevance to medical resource allocation | 2015 |
| 7 | Guiding Opinions on Carrying out the Pilot Work for the Construction of Medical Consortia | National Health and Family Planning Commission (abolished) | Substantial overlap with included policy P10 | 2016 |
| 8 | Outline of the National Informatization Development Strategy | General Office of the Central Committee of the Communist Party of China; General Office of the State Council | Mainly focused on informatization strategy; medical resource allocation only briefly mentioned | 2016 |
| 9 | Guiding Opinions on Promoting the Healthy Development of the Pharmaceutical Industry | General Office of the State Council | Mainly focused on pharmaceutical industry development; medical resource allocation only incidentally mentioned | 2016 |
| 10 | Notice on Issuing the Key Tasks for Deepening the Reform of the Medical and Health System in 2016 | General Office of the State Council | Annual task-arrangement document; insufficiently substantive and independent for PMC-index coding | 2016 |
| 11 | Notice on Issuing the Thirteenth Five-Year Plan for Poverty Alleviation | the State Council | Mainly focused on poverty alleviation; medical resource allocation only marginally mentioned | 2016 |
| 12 | Notice on Issuing the Key Tasks for Deepening the Reform of the Medical and Health System in 2017 | General Office of the State Council | Annual task-arrangement document; insufficiently substantive and independent for PMC-index coding | 2017 |
| 13 | Opinions on the Division of Key Tasks among Departments for Implementing the Government Work Report (2018) | the State Council | Broad task-allocation document; medical resource allocation not developed as a core policy theme | 2018 |
| 14 | Outline of the Integrated Regional Development Plan for the Yangtze River Delta | Central Committee of the Communist Party of China, the State Council | Broad regional development plan; medical resource allocation mentioned only at the planning level | 2019 |
| 15 | Notice on Issuing the Key Tasks for Deepening the Reform of the Medical and Health System in the Second Half of 2020 | General Office of the State Council | Annual task-arrangement document; insufficiently substantive and independent for PMC-index coding | 2020 |
| 16 | Opinions on Supporting Zhejiang’s High-Quality Development and Building a Demonstration Zone for Common Prosperity | Central Committee of the Communist Party of China, the State Council | Mainly focused on regional development and common prosperity; medical resource allocation only incidentally mentioned | 2021 |
| 17 | Opinions on Promoting the High-Quality Development of Public Hospitals | General Office of the State Council | Mainly focused on public hospital development; medical resource allocation not developed as a principal theme | 2021 |
| 18 | Guiding Opinions on Improving the Long-Term Mechanism of Basic Medical Insurance Participation | General Office of the State Council | Mainly focused on insurance participation; medical resource allocation only marginally mentioned | 2024 |
| 19 | Opinions on Promoting High-Quality Urban Development | Central Committee of the Communist Party of China, the State Council | Mainly focused on urban development; medical resource allocation only briefly mentioned | 2025 |
| 20 | Opinions on Further Safeguarding and Improving People’s Livelihoods and Resolving the Urgent Concerns of the People | General Office of the Central Committee of the Communist Party of China; General Office of the State Council | Mainly focused on livelihood protection; limited substantive content on medical resource allocation | 2025 |

Note: Detailed exclusion criteria are presented in the main text. The “Reason for exclusion” column identifies the primary thematic focus of each excluded document or the specific exclusion criterion applied.
